# Supplementary material for: Engineering a Microphysiological Model for Regenerative Endodontic Studies
Source: Biology (Basel). 2024 Mar 28;13(4):221. doi: 10.3390/biology13040221 (PMC11048264; doi:10.3390/biology13040221)
Supplement: Supplementary file 1 [file biology-13-00221-s001.zip › biology-2902460-supplementary.pdf]

Supplementary Figure S1

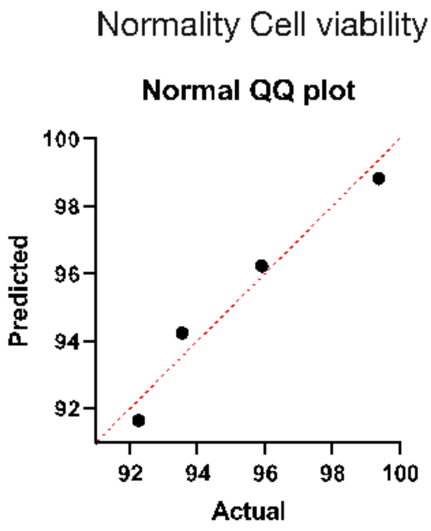

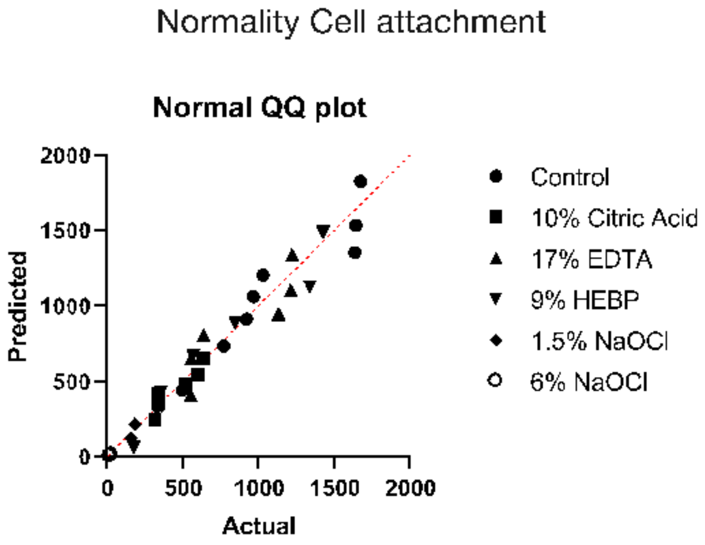

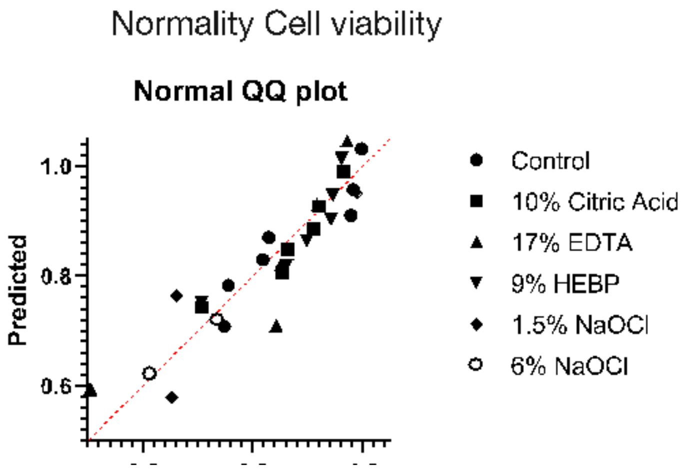

Supplementary Figure S1. Q-Q normal plots for 2D cell viability, 3D cell attachment and 3D cell viability.
